# Stability of Monkeypox Virus in Body Fluids and Wastewater

**DOI:** 10.3201/eid2910.230824

**Published:** 2023-10

**Authors:** Claude Kwe Yinda, Dylan H. Morris, Robert J. Fischer, Shane Gallogly, Zachary A. Weishampel, Julia R. Port, Trenton Bushmaker, Jonathan E. Schulz, Kyle Bibby, Neeltje van Doremalen, James O. Lloyd-Smith, Vincent J. Munster

**Affiliations:** National Institute of Allergy and Infectious Diseases, Laboratory of Virology, Hamilton, Montana, USA (C.K. Yinda, R.J. Fischer, S. Gallogly, Z.A. Weishampel, J.R. Port, T. Bushmaker, J.E. Schulz, N. van Doremalen, V.J. Munster);; University of California, Los Angeles, California, USA (D.H. Morris, J.O. Lloyd-Smith);; University of Notre Dame, Notre Dame, Indiana, USA (K. Bibby)

**Keywords:** mpox, human monkeypox virus, viruses, sexually transmitted infections, virus stability, surfaces, body fluids, United States

## Abstract

An outbreak of human mpox infection in nonendemic countries appears to have been driven largely by transmission through body fluids or skin-to-skin contact during sexual activity. We evaluated the stability of monkeypox virus (MPXV) in different environments and specific body fluids and tested the effectiveness of decontamination methodologies. MPXV decayed faster at higher temperatures, and rates varied considerably depending on the medium in which virus was suspended, both in solution and on surfaces. More proteinaceous fluids supported greater persistence. Chlorination was an effective decontamination technique, but only at higher concentrations. Wastewater was more difficult to decontaminate than plain deionized water; testing for infectious MPXV could be a helpful addition to PCR-based wastewater surveillance when high levels of viral DNA are detected. Our findings suggest that, because virus stability is sufficient to support environmental MPXV transmission in healthcare settings, exposure and dose-response will be limiting factors for those transmission routes.

Human mpox is an infectious zoonotic disease caused by monkeypox virus (MPXV) that was first discovered in 1958 in nonhuman primates in a laboratory setting (*1*). Even though exact animal reservoirs are unknown, small mammals are thought to maintain MPXV in West and Central Africa (*2*,*3*), where the virus is endemic (*4*) and periodic spillover into humans and limited onward transmission occur (*5*). Historically, MPXV cases have been identified sporadically outside of endemic regions, mostly related to travel, nosocomial contact, or contact with imported rodents (*6*). 

There are 2 known clades of MPXV: clades I (formerly Congo Basin clade) and II (formerly West Africa clade) (*7*). In May 2022, the largest known human outbreak of mpox began; this multinational outbreak is caused by clade IIb MPXV. On July 23, 2022, the World Health Organization declared the human mpox outbreak a Public Health Emergency of International Concern (*8*). Since then, >87,000 laboratory-confirmed cases have been reported, most outside of endemic regions. 

Human-to-human transmission of MPXV is likely to occur through direct contact or, potentially, fomites such as clothes, utensils, and bedding (*7*). During the ongoing outbreak, most cases have involved men who have sex with men (MSM). Sexual activity has been shown to be a likely route of transmission through skin-to-skin contact or sharing of body fluids. MPXV has been detected in a wide variety of samples including blood, saliva, urine, feces, semen, and skin, as well as rectal and oropharyngeal swab specimens (*9**,**10*). Environmental sampling detected low amounts of viable MPXV on household surfaces, even 15 days after initial discovery (*11*). In addition, MPXV genetic material has been detected in wastewater streams (*12*), prompting concern about risk of infection for wastewater workers or possible reverse spillover into populations (*13*). We evaluated stability in body fluids on surfaces and in wastewater of MPXV isolate hMPXV/USA/MA001/2022 (MA001), isolated in May 2022 from a human case-patient in Massachusetts, USA, and assessed the effectiveness of decontamination methods using chlorination. 

## Methods

We performed all experiments using 4.8 × 10^6^ plaque forming units (PFU)/mL clade II MPXV MA001 under maximum containment conditions at Rocky Mountain Laboratories (Hamilton, MT, USA). We propagated the virus in VeroE6 cells in Dulbecco modified Eagle medium (DMEM; Sigma-Aldrich, https://www.sigmaaldrich.com) supplemented with 10% fetal bovine serum, 1 mmol L-glutamine, 50 U/mL penicillin, and 50 μg/mL streptomycin (10% DMEM). We completed all experiments in triplicate at room temperature (21°C–23°C) unless otherwise indicated. We quantified MPXV using a plaque assay; limit of detection for all replicates was 2 PFU/mL. All experimental measurements are reported as medians across 3 replicates. We acquired human body fluids commercially from Lee BioSolutions Inc. (now Medix Biochemica USA Inc., https://www.leebio.com). Wastewater samples were collected from a municipal wastewater treatment plant in northern Indiana, USA, then shipped frozen overnight to Rocky Mountain Laboratories, where they were stored at –80°C until used as described elsewhere (*14*). 

### Stability of MPXV on Surfaces under Different Environmental Conditions

We evaluated the surface stability of MPXV MA001 on 15 mm polypropylene, AISI 316L alloy stainless steel disks, and cotton in conditions representing temperate fall (4°C/40% relative humidity [RH]), controlled room (21°C–23°C/40% RH), and tropical (28°C/65% RH) environments. We produced controlled environmental conditions in environmental chambers (MMM Group, https://www.mmm-medcenter.com) with protection from UV-B and UV-C exposure. After each environmental condition was established and maintained, we deposited 50 μL (7–10 drops) of MPXV stock containing 10^5^ PFU on the surface of a disk. At time of deposition (day 0) and 7 additional predefined timepoints (1, 3, 5, 7, 10, 15, and 20 days after deposition), we recovered deposited virus by rinsing with 1 mL of DMEM supplemented with 2% fetal bovine serum, 1 mmol L-glutamine, 50 U/mL penicillin, and 50 μg/mL streptomycin (2% DMEM) and froze the samples at –80°C until time of titration. 

### MPXV Stability in Human Body Fluids 

We measured stability of MPXV on surfaces by pipetting 50 μL of each body fluid containing 10^5^ PFU of MPXV on surface plastic or in solution containing 2.0 × 10^6^ PFU/mL (10^5^ PFU/50 μL) stored in a screw-top vial at 21°C/40% RH. To determine the stability of MPXV in body fluids we spiked blood, semen, serum, saliva, urine, and feces with MPXV MA001. To determine the stability of the virus in body fluids deposited on surfaces and allowed to dry naturally, we aliquoted 50 μL of each fluid containing 10^5^ PFU of MPXV onto a polypropylene disk and left them at 21°–23°C/40% RH. We recovered samples for each fluid at time of deposit and 1-, 3-, 5-, 7-, 10-, 15-, and 20-day timepoints by rinsing with 1 mL of 2% DMEM and froze the samples at −80°C until titrated. To determine the virus stability in solution, we initially prepared fluid containing 2.0 × 10^6^ PFU/mL (10^5^ PFU/50 μL). We stored solution samples (5 mL each) in screw-top vials at room temperature between sampling times. At time of deposition and 1-, 3-, 5-, 7-, 10-, 15-, and 20-day timepoints, we pipetted 50 μL of each fluid-virus mix into 1 mL of 2% DMEM and froze the samples at –80°C until time of titration. 

### MPXV Stability in Wastewater and Deionized Water

To assess the stability of MPXV in wastewater and deionized water, we diluted 50 µL of stock virus in 5 mL of wastewater (irradiated with 5 millirads to inactivate possible contaminants, 1:100) and deionized water (1:100 dilution) in triplicate. At time of deposition and 1-, 3-, 5-, 7-, 10-, 15-, and 20-day timepoints, we placed 100 µL of virus-spiked sample in 900 µL of DMEM supplemented with 2% DMEM and froze the samples at −80°C until time of titration. Physiochemical parameters of the wastewater have been reported elsewhere (*14*) 

### Wastewater Disinfection

To test the efficacy of free chlorine for disinfecting MPXV in wastewater, we diluted stock virus 100 times in wastewater and added 1.098 mL to each well in the top row of a deep-well 96-well plate. We took a sample from the solution before adding chlorine to obtain the initial virus concentration in the sample. For each concentration we added Acros Organics sodium hypochlorite (ThermoFisher, https://www.thermofisher.com) to 3 wells each to obtain initial doses of 0, 1, 5, or 10 parts per million (ppm). At initiation and 1-, 5-, 10-, 30-, and 60-minute timepoints, we added 100 µL samples of the wastewater solution to 100 µL of 0, 1, 5, and 10 ppm sodium thiosulfate solution to quench remaining free chlorine. We titrated the resulting solution and transferred it in total to 800 µL of DMEM supplemented with 2% DMEM. We froze samples at –80°C until time of titration. 

### Virus Quantification Using Endpoint Titration Plaque Assay

We thawed frozen samples and performed 10× serial dilutions. We added 250 μL of each dilution to a well of confluent Vero E6 cells in a 12-well plate and incubated them for 2 h. After 2 h, we added an additional 1 mL of 2% DMEM to each well. We incubated plates at 37°C with 5% CO_2_ for 4 d. On day 4, we removed the medium from the wells and replaced it with 10% formaldehyde for 10 min. After 10 min, we removed the formalin and replaced it with a 1% solution of crystal violet. The crystal violet remained on the cells for 10 min, at which point we removed it and rinsed the plates with water. After drying, we assessed the plates for plaques. We inferred individual titers and virus half-lives in a Bayesian framework (Appendix), modeling the plaque counts observed in titration wells as Poisson distributed, as reported elsewhere (*15*). 

## Results

**Figure 1:**
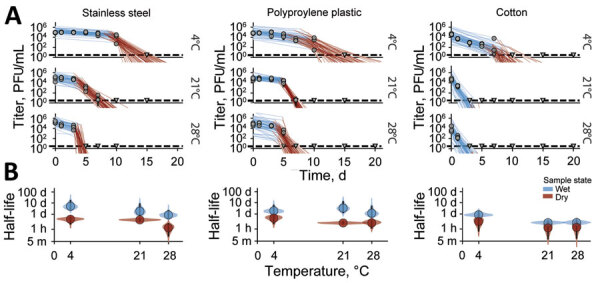
Monkeypox virus decay on cotton, polypropylene, and stainless steel under different environmental conditions. A) Regression lines showing predicted exponential decay of virus titers over time compared with measured (directly inferred) virus titers. Points show posterior median of measured titers; black lines show 95% credible intervals. Colored lines indicate random draws from the joint posterior distribution of exponential decay rate (negative of the slope) and intercept (initial virus titer), visualizing range of possible decay patterns for each experimental condition. Blue lines show virus titers during the inferred wet phase, when residual moisture remains visible on the surface; red lines show virus titers during the inferred dry phase, when evaporation has reached a state of quasi-equilibrium. The exact breakpoint was inferred from the data with a previous measurement from the last day of observable liquid. B) Inferred virus half-lives by surface and temperature condition. Dots show the posterior median half-life estimate and black lines show 68% (thick) and 95% (thin) credible intervals. Violin plots show the shape of posterior distribution. Blue show inferred virus half-lives on surfaces during wet phase and red on surfaces during dry phase.

In DMEM on surfaces, MPXV showed a biphasic pattern of initially slow, followed by rapid, decay. Because the transition in pace of decay typically occurred when all visible liquid had evaporated from the surface, consistent with observations for SARS-CoV-2 (*15*), we termed those periods the wet and dry phases. MPXV was less stable at higher temperatures, consistent with theoretical expectations (*15*) (Figure 1, panel A). It was more stable on stainless steel and polypropylene surfaces than on cotton, although recovering viable virus from a porous, absorbent surface like cotton may differ from recovery from nonporous, nonabsorbent surfaces, such as stainless steel (Figure 1, panel A). We calculated posterior median estimated half-lives (T_1/2_) (interquartile range [IQR] 2.5%–97.5%) for the wet and dry phases (Appendix Table). T_1/2_ on cotton during the dry phase could not be estimated for the 21°C–23°C room temperature and 28°C tropical conditions because we could detect no viable virus after the point of macroscopically observed drying of surfaces (Figure 1, panel A).

**Figure 2:**
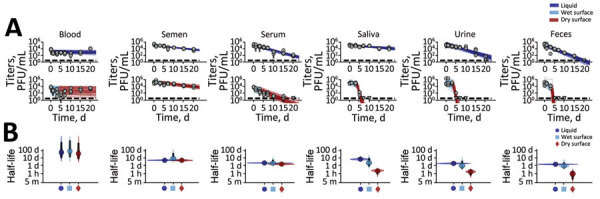
Monkeypox virus decay in human blood, semen, serum, saliva, urine, and feces solutions deposited on surfaces. A) Regression lines showing predicted exponential decay of virus titers over time compared with measured (directly inferred) virus titers. Points show posterior median measured titers; black lines show 95% credible intervals. Colored lines indicate random draws from joint posterior distribution of exponential decay rate (negative of the slope) and intercept (initial virus titer), visualizing range of possible decay patterns for each experimental condition. Top row shows experiments in bulk solution (liquid); bottom row shows experiments on surfaces. For surface experiments, light blue lines show the inferred titers during the wet phase, when visible residual moisture remains on the surface; red lines show the inferred dry phase, when evaporation has reached a state of quasi-equilibrium. The exact breakpoint was inferred from the data with a previous measurement from the last day of observable liquid. B) Inferred virus half-lives by condition and state. Dots show the posterior median half-life estimate and black lines show 68% (thick) and 95% (thin) credible intervals. Violin plots show the shape of posterior distribution. Dark blue show inferred virus half-lives in bulk solution, light blue on surfaces during wet phase, and red on surfaces during dry phase.

Next, we investigated the stability of MPXV in body fluids: blood, semen, serum, saliva, urine, and feces (Figure 2, panel A). We evaluated all matrices both on surfaces and in solution. Virus half-life showed no obvious differences between the wet and dry phases in blood, semen, and serum; half-lives during both phases were similar to half-lives in DMEM solution (Figure 2, panel B). In contrast, for saliva, urine, and feces on surfaces, virus half-lives were notably longer during the wet than the dry phase, and for all 3 secretions, half-lives were similar in solution (Figure 2, panel B). 

MPXV in blood and semen showed little or no detectable decay either in solution or on surfaces during the 20-day test period (Figure 2, panel A; Appendix Table 1). Results for blood on surface and in solution varied notably. T_1/2_ for blood in solution was 58.90 days (IQR 10.00–1,638.42 days) and on surfaces (dry phase) was 38.75 days (IQR 6.75–1,234.38 days). T_1/2_ for semen in solution was 4.63 days (IQR 3.94–5.70 days) and on surfaces (dry phase) was 4.57 days (IQR 3.35–7.09 days). MPXV in serum decayed over the test period, but with long half-lives of >1 days. T_1/2_ for serum in solution was 1.93 days (IQR 1.71–2.27 days) and on surfaces (dry phase) was 1.32 days (IQR 0.98–1.78 days) (Figure 2, panels A, B). 

MPXV had a long half-life in saliva, both in solution and on surfaces during the wet phase. T_1/2_ for saliva in solution was 6.49 days (IQR 4.72–10.75 days) (Figure 2, panel B, Appendix Table 1) and on surfaces (wet phase) was 2.05 days (IQR 0.66–9.84 days), but it decayed rapidly during the dry phase: T_1/2_ = 0.16 days (IQR 0.05–0.25 days). MPXV was less stable in urine and feces, but similar to results for some other body fluids, decay accelerated during the dry phase on surfaces (Figure 2, panel B). T_1/2_ for urine in solution was 1.69 days (IQR 1.35–2.17 days); on surfaces (wet phase), 0.86 days (IQR 0.32–4.10 days); and on surfaces (dry phase), 0.11 days (IQR 0.03–0.21 days). T_1/2_ for feces in solution was 1.28 days (IQR 1.07–1.53 days); on surfaces (wet phase), 0.76 days (IQR 0.35–2.51 days); and on surfaces (dry phase), 0.06 days (IQR 0.01–0.14 days). 

**Figure 3:**
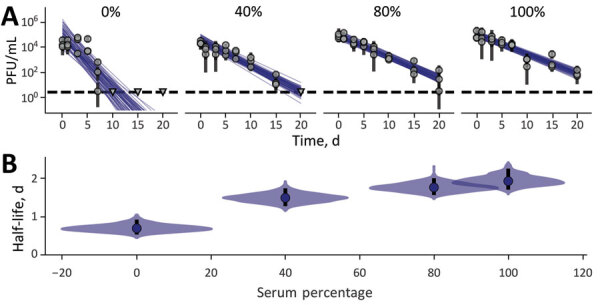
Monkeypox virus decay in different human serum dilutions in Dulbecco modified Eagle medium. A) Regression lines showing predicted exponential decay of virus titers over time compared with measured (directly inferred) virus titers. Points show posterior median measured titers; black lines show 95% credible intervals. Colored lines indicate random draws from joint posterior distribution of the exponential decay rate (negative of the slope) and intercept (initial virus titer), visualizing range of possible decay patterns for each experimental condition. B) Inferred virus half-lives by condition and state. Dots show posterior median half-life estimate and black lines show 68% (thick) and 95% (thin) credible intervals. Violin plots show the shape of posterior distribution of virus half-lives.

Overall, MPXV was consistently at least as stable in bulk liquid environments as on surfaces, especially wet surfaces. Stability on wet versus dry surfaces differed notably for MPXV in saliva, urine, and feces but not for MPXV in blood, semen, and serum. On the basis of those differences in decay patterns for MPXV and for other viruses, as reported elsewhere (*16*), we hypothesized that a highly proteinaceous environment provides protection against decay of the virus, perhaps particularly during and after evaporation of residual water following deposition. To investigate this hypothesis, we assessed stability of MPXV in solution incorporating increasing percentages (0%, 40%, 80%, 100%) of serum mixed with DMEM. Virus stability (measured in half-lives) monotonically increased as a function of the percentage of serum (Figure 3). 

**Figure 4:**
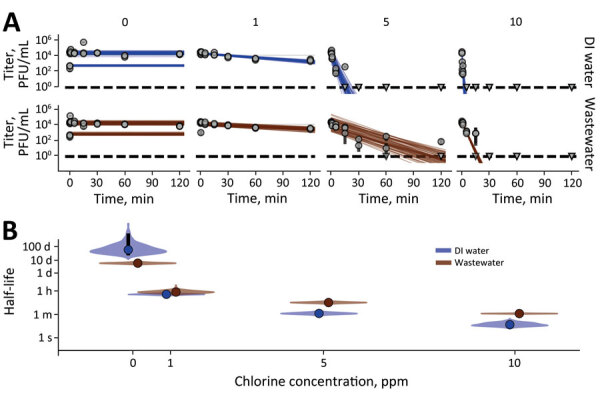
Monkeypox virus exponential decay and decontamination in wastewater and DI water. A) Regression lines showing predicted exponential decay of virus titer over time compared with measured (directly inferred) virus titers. Points show posterior median measured titers; black lines show 95% exponential decay rate (negative of the slope) and intercept (initial virus titer), visualizing range of possible decay patterns for each experimental condition. B) Inferred virus half-lives as a function of free chlorine concentration in parts per million. Violin plots show the shape of the posterior distribution of virus half-lives. Dots show credible intervals for posterior median half-life estimates and black lines show 68% (thick) and 95% (thin) credible intervals. Violin plots show the shape of posterior distribution. Dark blue show inferred virus half-lives in DI water and red in wastewater. DI, deionized; ppms, parts per million.

Finally, we determined the stability of MPXV and effectiveness of sodium hypochlorite to inactivate MPXV in wastewater and deionized water (Figure 4). In untreated deionized water, MPXV did not decay during the sampling period: T_1/2_ = 60.79 days (IQR 22.67–1078.62 days) (Figure 3). MPXV decayed to a meaningful level in wastewater, but with a half-life of multiple days (T_1/2_ = 5.74 [IQR 4.58–8.05] days) (Figure 3; Appendix Figure 1). MPXV rapidly became inactivated in deionized water with added sodium hypochlorite; T_1/2_ was 1.19 minutes (IQR 0.85–1.71 minutes) at 5 ppm free chlorine and 0.17 minutes (IQR 0.10–0.34 minutes) at 10 ppm. Higher chlorine concentrations were required for rapid inactivation of MPXV in contaminated wastewater samples: T_1/2_ of viable virus was 8.13 minutes (IQR 6.45–10.50 minutes) at 5 ppm chlorine and 1.17 minutes (IQR 1.05–1.28 minutes) at 10 ppm. Differences in required chlorine concentrations could be because of high free-chlorine consumption by the wastewater (*17*). These results suggest that MPXV is quite stable in untreated water, including wastewater, but that wastewater can be disinfected quickly, substantially reducing levels of viable virus.

## Discussion

Different studies investigating the stability of viruses of the genus *Orthopoxvirus* (family Poxiviridae) have arrived at similar conclusions as this study. Prolonged variola stability has been reported in scabs, vesicle and pustule fluids, lymph system, and purulent sores of patients (*18*). Also, investigations of variola in raw cotton and vaccinia in storm water and feces showed that a few virus particles may survive for long periods of time (*18**–**21*). Only a few studies have tested the stability of MPXV. An experimental study conducted on MPXV aerosol indicated the virus could remain viable in aerosol form for a prolonged period (*22*). Two other studies measuring stability and inactivation of MPXV showed the virus could be efficiently inactivated by alcohol- and aldehyde-based surface disinfectants. When World Health Organization‒recommended alcohol-based hand rub solutions were used to test disinfection, MPXV displayed greater stability than all other emerging or reemerging enveloped viruses (*23*,*24*). 

We found that MPXV indeed shows strong environmental persistence on surfaces and in solution. MPXV in some media (DMEM with human saliva, urine, and feces) showed clear biphasic decay on surfaces but not in others (blood, semen, and serum). The observed biphasic decay was indicative of stability kinetics differing from the virus initially deposited on surfaces in a liquid solution to virus remaining after macroscopic evaporation of the solution. Stability also varied depending on the various MPXV-containing fluids and wastewater we tested. MPXV persisting in clinical specimens (*25**–**27*) or tissues also suggested fluid-dependent rates of decay. More proteinaceous solutions, such as blood, serum, and semen, favored virus stability. We confirmed experimentally that the protective effect of serum was directly proportional to the concentration of serum. That finding was consistent with observations from other recent studies that environmental inactivation of viruses can be slowed by proteins in the solution and is strongly dependent on physicochemical properties of the medium (*16*). 

Environmental risk assessment has typically focused on properties of the ambient environment (temperature, humidity, surface type for fomite transmission, ventilation rate for airborne transmission). Taken together with previous work on other viruses, our results suggest that route and type of contamination should also be considered, because viability of viruses may also depend on the body fluids from which they are shed. That factor may partly account for variability in persistence of environmental MPXV contamination on different surfaces (*11*) and in discrepancies between the longevity of MPXV on cotton in this study compared with results from epidemiologic investigations in which exudate (vesicular or pustular fluids) provided more virus-protective environments (*18*). So far, of the many cases reported among healthcare workers, only a few have been occupationally acquired (*28**–**32*), suggesting that risk for workplace transmission of MPXV to healthcare workers is notably low. In addition, many environmental surfaces are regularly exposed to UV light and common household disinfectants, which decrease viral infectivity. Nevertheless, persistence of MPXV in the environment suggests that precautions are required to avoid environmental and nosocomial transmission, in particular in hospital settings. 

MPXV transmission and spread through sexual contact, especially among MSM, has been confirmed. That means long half-lives of viable MPXV in blood and semen increase risk of transmission through fluid exchange or skin-to-skin contact during sexual activity. Potential long-term viability in semen presents further implications for viral load in infected persons and for duration of infectiousness even after viral replication stops. Genetic material from other viruses, including Zika and Ebola, has been detected in semen months or years after initial infection, but how this relates to MPXV infectivity remains an open question (*33*). Estimated viral half-lives in our work are consistent with infectious MPXV remaining in semen for weeks after virus replication ends, as reported elsewhere (*34*,*35*). It should be noted however, that in patient blood, so far only DNA has been detected, and what roles blood and serum can play in MPXV transmission remains unclear (*36*). 

Our finding that MPXV can remain infectious for weeks in untreated wastewater raises the potential for risk of exposure among sanitation workers, peridomestic animals, and wildlife (*13*). Given the suspected role of rodents as reservoirs of MPXV, this possibility raises hypothetical concerns about zoonotic reservoirs becoming established in previously nonendemic countries. However, we emphasize that dilution and chemical disinfection can mitigate these risks. Because previous studies only tested for viral DNA (*12*,*37**–**40*), we suggest that testing for infectious MPXV could be a valuable complement to PCR-based wastewater surveillance when significant quantities of viral DNA are detected. 

In conclusion, our results suggest that MPXV stability is dependent on the surface, the environmental conditions, and the matrix of the virus. Overall, we found MPXV showed long half-lives in a variety of body fluids, both in bulk solutions and when deposited wet then allowed to dry on common clinical and residential surfaces, and half-lives approaching a week in untreated wastewater. Our findings suggest that, because virus stability is sufficient to support environmental or fomite transmission of MPXV, exposure and dose-response will be limiting factors for those transmission routes. 

Appendix Additional information about stability of monkeypox virus in body fluids, on surfaces, and in wastewater.
