# Supplementary material for: Stability of Monkeypox Virus in Body Fluids and Wastewater
Source: Emerg Infect Dis. 2023 Oct;29(10):2065–72. doi: 10.3201/eid2910.230824 (PMC10521604; doi:10.3201/eid2910.230824)
Supplement: Appendix — Additional information about stability of monkeypox virus in body fluids, on surfaces, and in wastewater. [file 23-0824-Techapp-s1.pdf]

2 Bayesian inference methods

2.1 Conceptual overview

As in our prior virus stability work [1]–[4], we infer individual titers and virus half-lives in a Bayesian framework. Such models can be used either to infer individual titers or to fit an exponential decay rate (equivalently, a half-life) to a set of samples taken at different timepoints. In the latter case, we jointly infer the decay rate and the individual sample titers, for maximally-principled error propagation. By also estimating individual titer values (without any assumptions about their relationship or the decay process), we are able to check the goodness-of-fit of the exponential decay model.

Our prior work quantified viable virus via an endpoint titration assay; here, we instead use a plaque assay. The underlying inferential models are the same except for the final step of the observation process, where a Poisson model for the observed number of plaques replaces the “single-hit” model [5] for the positive or negative status of an individual well.

When inferring virus decay rates, we typically describe models in terms of exponential decay rates of viable virus λ, which has units of $\log_b$ viable virus per unit time, for some base b. It is typically easier to interpret the mathematically equivalent half-life values h, given by:

$$h = \frac{\log_b(2)}{\lambda} \tag{1}$$

Here, we measure titers in log base 10, so:

$$h = \frac{\log_{10}(2)}{\lambda} \tag{2}$$

We typically place priors on log half-lives $\log(h)$ rather than on decay rates λ, and then calculate the implied λ.

2.2 Notation

In the text that follows, we use the following mathematical notation.

2.2.1 Logarithms and exponentials

$\log(x)$ denotes the logarithm base e of x (sometimes called $\ln(x)$). We explicitly refer to the logarithm base 10 of x as $\log_{10}(x)$. $\exp(x)$ denotes e^x.

2.2.2 Probability distributions

The symbol $\sim$ denotes that a random variable is distributed according to a given probability distribution. So for example

$$X \sim \text{Normal}(0, 1)$$

indicates that the random variable X is normally distributed with mean 0 and standard deviation 1.

We parameterize normal distributions as:

$$\text{Normal}(\text{mean}, \text{standard deviation})$$

We parameterize positive-constrained and negative-constrained normal distributions (i.e. truncated Normal distributions with lower limit 0 and upper limit ∞, or lower limit $-\infty$ and upper limit 0, respectively) as:

$$\text{PosNormal}(\text{mode}, \text{standard deviation})$$

$$\text{NegNormal}(\text{mode}, \text{standard deviation})$$

More generally, we parameterize truncated normal distributions with arbitrary lower and upper limits of truncation as:

$$\text{TruncNormal}(\text{mode}, \text{standard deviation}, \text{lower limit}, \text{upper limit})$$

We parameterize Poisson distributions as:

$$\text{Poisson}(\text{mean})$$

2.3 Poisson observation model

Given a sample with underlying virus concentration v_i in units of $\log_{10}$PFU/volume diluted by a $\log_{10}$ dilution factor d_i and then plated onto susceptible cells in a volume p_i, we model the observed plaque count y_i as:

$$y_i \sim \text{Poisson}(p_i 10^{v_i - d_i} + f) \tag{3}$$

where f is an inferred or assumed false hit rate that can be used to model false positive plaques.

That is, we treat the observed plaque count as Poisson distributed with a mean given by the underlying virus concentration, the volume plated, the dilution performed, and any sources of error that could lead to spurious "plaques".

Here, for simplicity, we assume a fixed negligible false hit rate of 10^{-20}. It is both unbiological and impractical to use a true zero false hit rate ($f = 0$), as this makes Markov Chain Monte Carlo inference less numerically stable.

2.4 Titer inference

To infer individual titers, we use a weakly informative Normal prior for the true virus concentrations v_i in units of PFU/mL:

$$v_i \sim \text{Normal}(3, 3) \tag{4}$$

We then apply the Poisson plaque assay model described in section 2.3 above.

2.5 Fluid half-life inference

We infer half-lives of infectious virus in various bulk liquid media by adapting a model previously previously described [2], [4], which allows us to account for variation in initial virus concentration, among other sources of experimental error.

There are multiple experimental conditions: the various liquid media tested. These include authentic human secretions including blood, saliva, and serum at several distinct concentrations, as well as deionized water and wastewater subjected to several distinct levels of chlorination.

Broadly, our model assumes that each replicate begins with some initial concentration of viable virus $v(0)$, which then decays exponentially at some rate λ, so that at time t, we have

$$\log_{10}[v(t)] = \log_{10}[v(0)] - \lambda t \tag{5}$$

To model our experiments, we need to account for the fact that we performed multiple replicates for each experimental condition. Each of these replicates might have begun with different initial virus concentrations. Additionally, individual samples taken over time and plaque counts derived from those samples might be over-dispersed relative to the ideal predicted Poisson counts (due to processes of sampling, plating, infection, and counting processes not being mathematically ideal).

We use hierarchical modeling to handle the potential differences in initial virus concentration among replicates. For each replicate k of experimental condition j, we model the initial $\log_{10}$ concentrations of viable virus $\log_{10}[v_{0jk}]$ as Normally distributed about an inferred condition-specific mean initial concentration $\log_{10}[\bar{v}_{0j}]$, with an inferred condition-specific standard deviation σ_{v0j}:

$$\log_{10}[v_{0jk}] \sim \text{Normal}(\log_{10}[\bar{v}_{0j}], \sigma_{v0j}) \tag{6}$$

Across all replicates, viable virus then decays exponentially at an inferred condition-specific rate λ_j. So we predict that if a sample v_{ijk} is taken for replicate k of condition j at a time t_{ijk}:

$$\log_{10}[v_{ijk}] = \log_{10}[v_{0jk}] - \lambda_j t_{ijk} + \sigma_{vj}\epsilon_{ijk} \tag{7}$$

where:

$$\epsilon_{ijk} \sim \text{Normal}(0, 1) \tag{8}$$

The Normally distributed errors ϵ_{ijk} represent deviations from ideality / potential over-dispersion of plaque counts. The condition-specific scaling factor σ_{vj} represents an inferred degree of deviation from ideality. In particular, note that this is equivalent to stating:

$$\log_{10}[v_{ijk}] \sim \text{Normal}(\log_{10}[v_{0jk}] - \lambda_j t_{ijk}, \sigma_{vj}) \tag{9}$$

Any observed plaque count(s) for y_{ijkl} are then modeled as Poisson distributed per equation 3:

$$y_i \sim \text{Poisson}(p_{ijk} 10^{v_{ijk} - d_{ijk}} + f) \tag{10}$$

In practice we typically have a single count y_{ijk} for each sample v_{ijk}, corresponding to the first dilution at which plaques were countable.

We use the following priors.

Log half-lives $\log(h_j)$ for each experimental condition j, where h_i has units of days:

$$\log(h_j) \sim \text{Normal}(\log[0.1], \log[20]) \tag{11}$$

Mean initial $\log_{10}$/PFU/mL virus titers $\log_{10}[\bar{v}_{0j}]$ for each experimental condition j:

$$\log_{10}[\bar{v}_{0j}] \sim \text{Normal}(3, 2) \tag{12}$$

Condition-specific standard deviations σ_{v0j} for the Normally-distributed replicate $\log_{10}$ initial virus concentrations:

$$\sigma_{v0j} \sim \text{PosNormal}(1, 0.25) \tag{13}$$

Condition-specific standard deviations σ_{vj} for the predicted titer errors:

$$\sigma_{vj} \sim \text{PosNormal}(0, 0.5) \tag{14}$$

2.6 Surface half-life inference

We modeled decay on surfaces analogously to how we modeled decay in bulk fluid form (section 2.5), with the additional detail that we allowed for biphasic decay to take account of potential effects of medium evaporation [3].

In particular, we allowed each surface experiment to have a inferred breakpoint time w_j, during which the half-life switches from a "wet-phase" half-life h_{wj} to a "dried-phase" half-life h_{dj}. We placed a Truncated Normal prior on the w_j on the interval between the last day in which the sample was observed to be macroscopically wet t_w and the first day it was observed to be macroscopically dry t_d, with a mode at the midpoint $(t_d - t_w)/2$, and a standard deviation of 2. In practice, this is very similar to a uniform prior on the interval (t_w, t_d).

$$w_j \sim \text{TruncNormal}\left(\frac{t_d - t_w}{2}, 2, t_w, t_d\right) \tag{15}$$

Until w_j, virus then exponentially at an inferred condition-specific rate $\lambda_{wj} = \frac{\log_{10}(2)}{h_{wj}}$ and afterward at an inferred condition-specific rate $\lambda_{dj} = \frac{\log_{10}(2)}{h_{dj}}$. So we predict that if a sample v_{ijk} is taken for replicate k of condition j at a time t_{ijk}:

$$\log_{10}[v_{ijk}] = \begin{cases} \log_{10}[v_{0jk}] - \lambda_{wj} t_{ijk} + \sigma_{vj}\epsilon_{ijk} & t_{ijk} \leq w_j \\ \log_{10}[v_{0jk}] - \lambda_{wj} w_j - \lambda_{dj}(t_{ijk} - w_j) + \sigma_{vj}\epsilon_{ijk} & t_{ijk} > w_j \end{cases} \tag{16}$$

The observation process was then the same as for titer inference and fluid half-life inference.

To parameterize the half-lives during the two phases in a principled way while avoiding identifiability issues, we placed a normal prior on the log wet-phase half-life h_{wj}, and the modeled the log dried-phase half-life h_{dj} as offset from h_{wj} by some inferred offset $o_j = \log(h_{dj}) - \log(h_{wj})$. We placed a negative-constrained normal prior on o_j(that is, we assumed dried-phase half-lives were shorter than wet-phase half-lives, consistent with prior empirical and thereotical work [3]:

$$\log(h_{wj}) \sim \text{Normal}(\log(0.25), \log(20)) \tag{17}$$

$$o_j \sim \text{Normal}(\log(0.25), \log(20)) \tag{18}$$

where

$$\log(h_{dj}) = \log(h_{wj}) + o_j \tag{19}$$

All other aspects of the model, including prior distribution choices, were identical to the fluid half-life inference model, with the except that we modeled condition-specific standard deviations σ_{vj} for the predicted titer errors as potentially larger, since surface experiments are noisier:

$$\sigma_{vj} \sim \text{PosNormal}(0, 1) \tag{20}$$

3 Code and data

All code and data needed to reproduce our analyses is archived on Github (https://github.com/dylanhmorris/mpox-stability) and Zenodo (https://example.com), and licensed for reuse, with appropriate attribution and citation.

Supplementary Appendix References

[1] R. J. Fischer, D. H. Morris, N. van Doremalen, *et al.*, "Effectiveness of N95 respirator decontamination and reuse against SARS-CoV-2 virus," *Emerging Infectious Diseases*, vol. 26, no. 9, p. 2253, 2020.

[2] A. Gamble, R. J. Fischer, D. H. Morris, C. K. Yinda, V. J. Munster, and J. O. Lloyd-Smith, "Heat-treated virus inactivation rate depends strongly on treatment procedure: Illustration with SARS-CoV-2," *Applied and Environmental Microbiology*, vol. 87, no. 19, e00314–21, 2021.

[3] D. H. Morris, K. C. Yinda, A. Gamble, *et al.*, "Mechanistic theory predicts the effects of temperature and humidity on inactivation of sars-cov-2 and other enveloped viruses," *eLife*, vol. 10, e65902, 2021.

[4] T. Bushmaker, C. K. Yinda, D. Morris, *et al.*, "Comparative Aerosol and Surface Stability of SARS-CoV-2 Variants of Concern," *Emerging Infectious Disease journal*, vol. 29, no. 5, p. 1033, 2023, ISSN: 1080-6059. DOI: 10.3201/eid2905.221752. [Online]. Available: https://wwwnc.cdc. gov/eid/article/29/5/22-1752_article.

[5] C. Brownie, J. Statt, P. Bauman, *et al.*, "Estimating viral titres in solutions with low viral loads," *Biologicals*, vol. 39, no. 4, pp. 224–230, 2011.
